# Social dimensions as explanatory approaches for the development of chronic pain: a meta-ethnography of qualitative studies

**DOI:** 10.1186/s12939-025-02560-w

**Published:** 2025-07-07

**Authors:** Sarah Potthoff, Dominik Koesling, Claudia Bozzaro

**Affiliations:** Institute for Ethics, History, and Theory of Medicine, University Münster, Von-Esmarch-Straße 62, Münster, 48149 Germany

**Keywords:** Chronic pain, Social dimension, Discrimination, Marginalization, Stigmatization, Poverty, Gender inequity

## Abstract

Chronic non-malignant pain – especially low back pain and headache disorders – is an increasingly common condition affecting many people globally. The biopsychosocial model of pain is widely used in the field of chronic pain research and considers the complex etiologies of chronic pain. Although the biopsychosocial model is well accepted, most studies address biomedical and psychological aspects, while neglecting the social dimension. The aim of our meta-ethnography was to systematically collect and analyze qualitative studies that explored social dimensions as explanatory approaches for chronic pain onset and progression. We used meta-ethnography guidelines and conducted systematic searches in three databases (PubMed, Web of Science, CINAHL) in November 2023, and an update in November 2024. Critical appraisal of included studies was performed using the JBI critical appraisal checklist for qualitative research. We included twenty-seven studies and developed eight categories of social dimensions that contribute to explaining chronic pain. These third order constructs were gender relations and gender inequity, stigmatization, discrimination, and marginalization based on social identity, adversities, harsh living and working conditions, high expectations regarding work or school, cultural and religious beliefs and values, loneliness, and lifestyle factors. Our findings help confirm that there are relevant social dimensions that contribute to the chronicity of pain. Addressing these dimensions is necessary for adequate understanding of chronic pain’s complexity and preventing chronic pain onset and progression.

## Introduction

Chronic pain is an increasingly common condition affecting many people globally, especially low back pain and headache disorders [1]. Approximately 20% of the population in Canada [55], and the United States [41, 62] suffer from chronic pain. The prevalence is even higher in other world regions such as in Africa [39], Asia [37], and Europe [56]. The International Association for the Study of Pain (IASP) defines pain as “an unpleasant sensory and emotional experience associated with, or resembling that associated with, actual or potential tissue damage” and this experience is “always a personal experience” ([49], p. 1977). Chronic pain is generally defined as persistent or recurrent pain that lasts longer than three months and develops into a long-term condition [57].

Scholars investigating chronic pain typically refer to the biopsychosocial model of pain. This model recognizes the complex etiologies of chronic pain including biomedical, psychological, and social influencing factors [20, 36]. However, most research in this area still relies on clinical and biomedical approaches that do not sufficiently take into account a critical theoretical understanding of the social [50, 61]. The social is often equated with the subjective/individual and subsumed under the category of the psychosocial, which is viewed exclusively from a psychological and not a sociological perspective [22, 33]. Some scholars have even criticized the biopsychosocial model itself as lacking theoretical foundations in its analysis of the social [53]. The social, as grounded in subjective experiences and at the same time embedded in the context of socio-structural contexts, still seems to be neglected in research into the causes of chronic pain. Quantitative epidemiological studies investigating causes of chronic pain increasingly consider social factors in addition to biomedical ones and have pointed to the prevalence of chronic pain by gender [7, 14], marginalized identities, and socioeconomic inequality [35, 48]. However, the focus of these studies is on the social factors that are objective, measurable or quantifiable. They do not address more subtle forms of societal interactions like e.g. forms of recognition or exclusion [32]. They further do not reflect the patient experience perspective.

Therefore, our meta-ethnography aimed to systematically collect and analyze qualitative studies that investigated social dimensions as explanatory approaches for the development of chronic pain. We included studies from around the world with people directly affected by chronic pain, their family members and friends, and healthcare practitioners. We excluded studies that discussed the social merely as an individual psychosocial phenomenon from a psychological perspective. Our meta-ethnography answers the question: Which social dimensions can be developed from previous qualitative studies on social dimensions as an explanatory approach for the onset and progression of chronic pain?

## Methods

We chose meta-ethnography as a method to synthesize qualitative studies on chronic pain because the purpose of this review was to generate theoretical knowledge about social dimensions as an explanation for chronic pain from existing empirical studies. Since its introduction in the 1980 s in education research by Noblit and Hare [43], meta-ethnography has been a widely used approach to synthesize qualitative studies in the health sciences [58]. While some qualitative synthesis approaches are primarily descriptive in nature, meta-ethnography is an interpretive approach that seeks not only to synthesize evidence on a topic of interest but also to arrive at new interpretations of that topic. The reviewers need to reinterpret primary participant quotes (first order constructs) as well as themes and concepts developed by the authors of the primary studies (second order constructs). The aim of the method is to generate new, higher-order interpretations (third order constructs) that go beyond the primary findings of the included studies. Accordingly, this method is particularly useful for developing conceptual models and theories of the investigated phenomenon [11, 21, 51].

### Inclusion and exclusion criteria

To guide our work, we developed a review protocol in which we established the following inclusion criteria:

- Content-related criteria:
  social dimensions as an explanation for chronic pain
- Information sources:
  original studies
- Languages:
  German and English
- Year of publication:
  until November 2024
- Origin of publications:
  worldwide
- Quality:
  eligible according to JBI critical appraisal checklist for qualitative research [30]

We excluded studies based on the following criteria:

- articles on malignant chronic pain
- studies that used exclusively quantitative methods

### Information sources and search strategy

To cover the perspectives of different disciplines, we selected three databases relevant to the topic: PubMed, Web of Science, and CINAHL. Table 1 shows our search strategy with the adapted algorithms. SP conducted the primary search for articles in November 2023 and conducted an update search in November 2024 using the same algorithms.

**Table 1:** Search strategy

Databases	Algorithm	Records (Nov. 2023)	Records (Nov. 2024)
CINAHL	("chronic pain") AND ("qualitative") AND (“social”);	168	188
	(“chronic pain”) AND (“qualitative”) AND (“society”);	22	22
	("chronic pain") AND (“social”) AND (“explanation”)	13	13
PubMed	("chronic pain") AND ("qualitative") AND (“social”);	356	418
	(“chronic pain”) AND (“qualitative”) AND (“society”);	40	47
	("chronic pain") AND (“social”) AND (“explanation”)	20	24
Web of Science	("chronic pain") AND ("qualitative") AND (“social”);	258	311
	(“chronic pain”) AND (“qualitative”) AND (“society”);	70	78
	("chronic pain") AND (“social”) AND (“explanation”)	12	15
Total		959	157
Total together			1,116

### Screening process and quality assessment

**Fig. 1:**
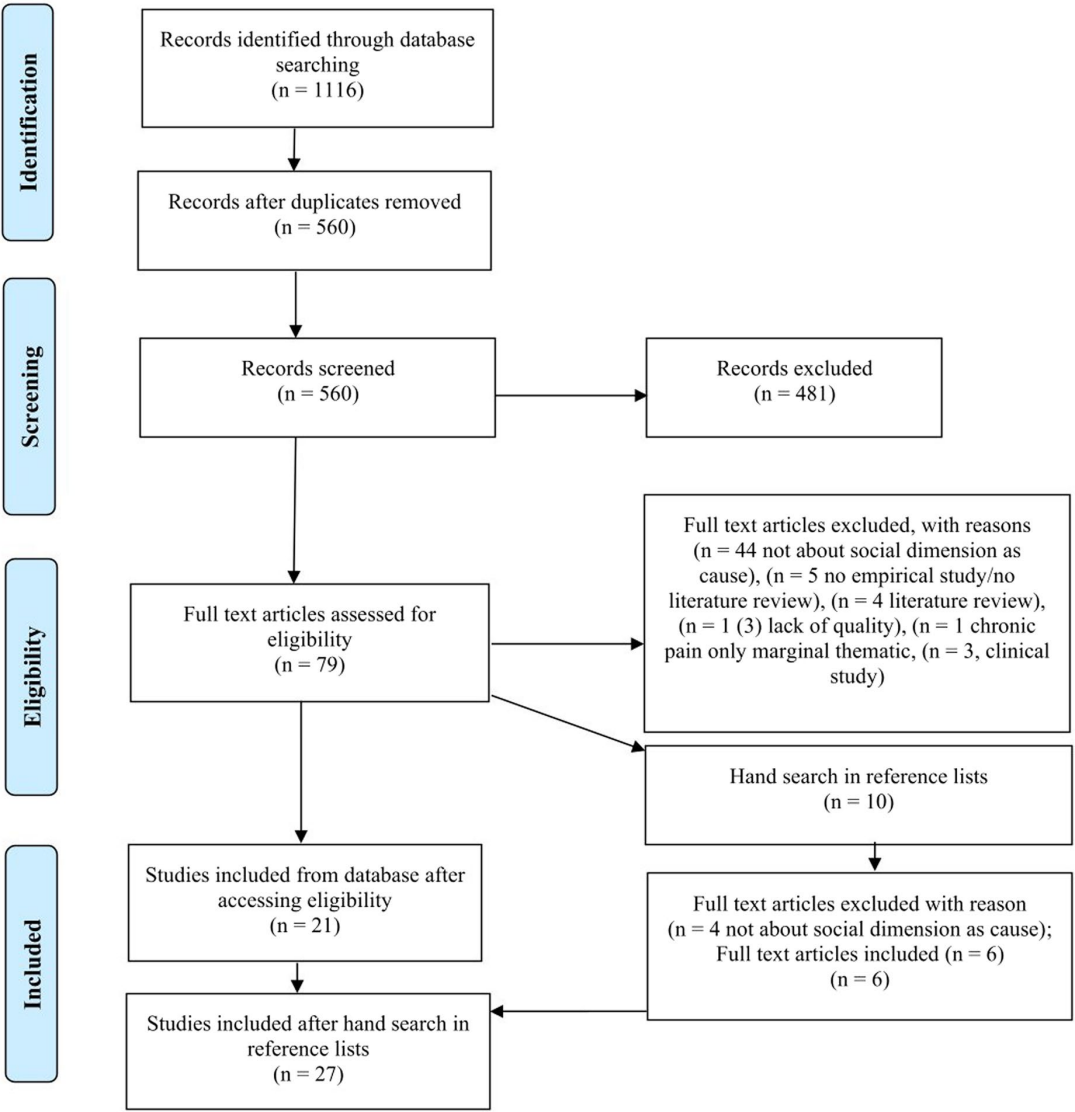
PRISMA flow diagram

We reduced the total number of records from 1,116 to 560 by excluding duplicates. SP screened all abstracts and titles for eligibility and two researchers SP and DK independently reviewed the abstracts and titles of 103 (11,5%, random sample) articles to ensure that the same publications were included and excluded. This resulted in 79 full texts, which SP checked for their eligibility and quality. For the critical appraisal of the quality, we used the JBI critical appraisal checklist for qualitative research [30]. Additionally, DK reviewed 14 full texts for which there were uncertainties regarding inclusion or exclusion to ensure validity. SP and DK discussed and solved any discrepancies and made a joint decision regarding inclusion and exclusion. This screening process resulted in 21 full texts being included in the review. In addition, SP checked the reference lists of the 21 included full texts for further relevant articles, which led to the inclusion of 6 further articles. We therefore included a total of 27 studies in this review. Figure 1 shows the entire review process as a PRISMA flow chart [44].

### Data extraction and data synthesis

Following the guidance for conducting a meta-ethnography SP and DK analyzed the 27 included studies [11, 21, 51]. After phase 3, “reading the studies,” SP transferred the studies into the software program MAXQDA 2020 to proceed to phase 4, “determining the relationships between studies,” phase 5, “translating the studies into one another,” and phase 6, “synthesizing translations.” SP started by synthesizing relevant characteristics of the studies in an Excel spreadsheet. Table 2 provides an overview of these characteristics. SP then inductively coded the results section of each study. In the next step, SP and DK compared and refined the codes from the different studies to identify common, recurring, and overarching categories. This reinterpretation resulted in the development of eight main categories. We present these below with references to first and second order themes and concepts of participants, and of authors of the primary studies.

**Table 2:** Overview of the characteristics of the included studies

Authors and year	Journal	Country of study	Research question(s)/aim	Study sample	Data collection method	Data analysis method	Study results
Abudoush et al. [2]	Frontiers in Psychology	UK, Jordan	This study aimed to explore the attentional experiences, coping mechanisms and suggestions for treating chronic pain among Arabic-speaking individuals in Jordan and Great Britain	51 Arabic-speaking individuals from GB and Jordan with chronic pain who had just completed two attention tasks	semi-structured face-to-face interviews	inductive framework thematic approach	Factors contributing towards developing or exacerbating chronic pain varied and included psychological (i.e., mood disturbance), contextual (i.e., job/work requiring attention) and social factors (i.e., social support)
Allen et al. [4]	BMC Family Practice	Canada	This study aimed to understand the experience of chronic pain among female Survival Sex Workers in Vancouver's downtown eastside	11 individuals self-identified as a current or former Survival Sex Worker and who had a chronic pain experience	semi-structured face-to-face interviews	exploratory qualitative analysis	This study builds on existing knowledge by suggesting that chronic pain in the setting of addictions and marginalization may be a symptom of more complex concerns associated with polysubstance use, trauma, poverty and stigma, in addition to mental and physical health issues
Arman et al. [5]	Qualitative Health Research	Sweden	This aimed to understand the lived experience of women with chronic pain from a caring science and gender perspective	21 women who had undergone a multimodal rehabilitation program for people with chronic pain	semi-structured face-to-face interviews	hermeneutical approach	The analysis revealed intertwined experiences of overperformance, loneliness, pain, and exhaustion. Women’s experience of an overwhelming life situation and the significance of mutual dependency seem to be central to health and suffering in women with chronic pain
Baum et al. [6]	PLOS ONE	Somali region of Ethiopia	This study aimed to explore the perceptions and notions of chronic pain among Somali pastoralists in the Somali region of Ethiopia	20 female and male Somali pastoralists with chronic pain	semi-structured face-to-face interviews	framework method	The analysis suggested that participants’ perceptions of their chronic pain can be clustered into six different themes: Pain as a symptom of harsh daily life, pain descriptions and dimensions, temporality of pain, stigma and stoicism, mediating role of spirituality; and impact of pain on daily life activities
Black et al. [8]	Patient Education and Counseling	Worldwide (online)	This study aimed to explore understanding, confusion, and gaps in knowledge about fibromyalgia among those who report a fibromyalgia diagnosis	38 participants who self-reported a previous diagnosis of fibromyalgia	online survey with open-end questions	thematic analysis	Themes that arose from analysis were being unsure of the cause of the fibromyalgia, frustration and confusion about the random/variable nature of symptoms and flares, feeling that their condition was invisible, and desiring more information on available treatments
Brady et al. [9]	Rheumatology Advances in Practice	Australia	This study aimed to explore the ethnocultural influences on the chronic pain experience in three culturally and linguistically diverse communities in Australia	41 individuals who self-identified as Mandaean, Assyrian or Vietnamese living with chronic pain	6 focus groups	inductive thematic analysis	Themes of ethnocultural identity and migrant status were intertwined in the unique explanatory model of pain communicated for each community
Brady et al. [10]	Pain Medicine	Australia	This study aimed to identify how multiple social identities intersect to account for the unequal distribution of the burden of chronic pain	41 individuals who self-identified as Mandaean, Assyrian or Vietnamese living with chronic pain	6 focus groups	inductive, intersectional methodology	The interaction between a patient with chronic pain from a culturally and linguistically diverse background and the health system is influenced by four identified social identities that interact to create relative positions of disadvantage for the patient within the health system and with health care providers. The social identities identified were ethnoculture, social class, migration status, and gender
Campeau [13]	Medical Humanities	USA	This study asked how Somali women understand pain and pain management	15 Somali refugee women; 3 health educators	15 semi-structured face-to-face interviews; 2 focus groups; participant observations from 15 chronic pain listening sessions	grounded theory methodology (Charmaz)	Four frameworks emerged through which participants experienced chronic pain: pain as a symptom of exile; pain and the strength to bear pain as issues of faith; medicine as powerful, curative and fluid; medical discrimination and exclusion
Clottey et al. [15]	Rural and Remote Health	USA	This study aimed to explore grandparent caregiving among rural, low-income, African American grandmothers in a community in the American South, and to identify challenges to health that arose in that context	12 African American grandparent caregivers who had primary responsibility for the care of coresident grandchildren	semi-structured face-to-face interviews	content analysis	Rural African American grandparent caregivers faced a range of challenges to health
Cousin et al. [17]	Advanves in Nursing Science	USA	This study aimed to identify the ways in which urban-dwelling Black older women displayed strength while living with chronic osteoarthritis pain	9 rural- and urban-dwelling Black older women	semi-structured face-to-face interviews	secondary qualitative analysis; descriptive phenomenological approach	Their “herstories” parallel the five characteristics of the Superwoman Schema/Strong Black Woman. Two additional characterizations emerged: spiritual submission for strength and code-switching to suffering Black woman; these may be unique to Black Americans with pain
Dassieu et al. [18]	Social Science & Medicine	Canada	This study aimed to understand how the experience of illicit drug use shapes the chronic pain experience	25 individuals who used street opioids and/or cocaine (with or without any other drug) and had suffered from chronic pain	semi-structured face-to-face interviews	grounded theory methodology (Corbin, Strauss; Glaser, Strauss)	Social deprivation and drug use increased PWUD's exposure to a wide range of health issues including chronic pain. Even when intense, pain was often described as peripheral in their life given their many other problems. They experienced double stigmatization due to the cumulation of two socially devalued statuses, “drug addicts” and “chronic pain sufferers.” Their attempts to avoid stigma included valuing their toughness/endurance and pursuing physical activities despite the pain
Eccleston et al. [19]	Social Science & Medicine	UK	This study asked how chronic pain patients and pain professionals make sense of the causes of chronic pain	16 chronic pain patients and 16 pain professionals	Q-sorting procedure	Q-factor analysis, Q-methodology	It is argued that when pain is no longer useful as a symptom, identity is challenged, weakened and at risk for both chronic pain patients and pain professionals
Furness et al. [23]	Health Psychology Open	UK	This study aimed to investigate in detail this highly significant aspect of the illness narrative of people with fibromyalgia	596 people with fibromyalgia	mixed-methods online survey	inductive thematic analysis	Themes were bodily assault, ill-health, and change; emotional trauma and distress; stress and vulnerability; and explaining and authenticating fibromyalgia
Hallberg & Carlsson [25]	Scandinavian Journal of Caring Sciences	Sweden	This study aimed to describe, from the perspective of women with fibromyalgia, their experiences and beliefs of the pain and its origin and how the pain affects family and social life	22 women with fibromyalgia	semi-structured face-to-face interviews	grounded theory methodology (Glaser, Strauss)	The first of the two core concepts, psychosocial vulnerability, comprised the categories: traumatic life history, over-compensatory perseverance, pessimistic life view, and unsatisfying work situation. The second core concept, maintaining forces, consisted of the categories professional care, pain benefits and family support, which seem to contribute to the persistence of pain
Hervik et al. [27]	Explore	Norway	This study aimed to gain subjective information regarding aspects in daily life, in order to answer the research question “What is life like with a chronic headache?”	16 patients who suffered from chronic headaches	semi-structured face-to-face interviews	thematic analysis	The majority believed that psychological and/or physiological trauma was the reason for their headaches In addition, everyday life events that are not normally considered major trauma triggered and maintained symptoms
Høie et al. [28]	BMC Nursing	Norway	This study aimed to explore how school nurses explain and experience the everyday pain of adolescents	17 school nurses	5 focus groups	qualitative content analysis	The experience of school nurses with adolescents’ pain in everyday life was mainly that pain is a social, physical, and psychological phenomenon. School nurses experienced that everyday pain reflects: high expectations, difficult relationships and traumatic experiences and an unhealthy lifestyle
Horment-Lara et al. [29]	Musculoskeletal Science and Practice	Chile	This study aimed to explore the beliefs of women with non-specific chronic low back pain in terms of nature of symptoms, fears associated with pain, expectations for recovery, family, social and work-related limitations, and perceived self-efficacy	10 women with non-specific chronic low back pain	semi-structured face-to-face interviews	deductive thematic analysis	Maladaptive beliefs about pain leading to fearful attitudes and low expectations for recovery. These beliefs seemed to perpetuate pain and limit engagement in daily tasks and meaningful activities. Some of these beliefs were associated with information provided by healthcare professions. Despite having maladaptive beliefs, women perceived themselves as self-effective
Locher et al. [34]	Pain Reports	Switzerland	This study aimed to explore pain concepts of Swiss pediatricians by means of a qualitative analysis	233 clinically active Swiss pediatricians	cross-sectional online survey; case vignette	qualitative structuring content analysis	Most pediatricians reported the belief that psychological factors explained the pain. However, when explaining the pain to the patient, biological factors were reported most often
Mustafa et al. [40]	Canadian Journal of Pain	Canada	This study aimed to explore immigrant Indian women’s chronic pain experiences in Canada and aimed to enhance the understanding of those experiences by creating a visual opportunity for them to share their stories	12 immigrant Indian women	captured photographs; semi-structured interviews	thematic analysis, phenomenological reflection	Three themes emerged from the analysis: bodies in pain, traversing spaces including immigration, and pain management methods. Findings revealed that women’s representations of pain were shaped by a clash between culturally shaped gender role expectations and changing gender norms due to immigration processes
Nigol et al. [42]	Rural and Remote Health	Australia	This study aimed to explore conceptualizations and beliefs about pain held by the Nepali-speaking Bhutanese community in rural and regional Australia	22 people from the Nepali-speaking Bhutanese community in regional Australia	focus groups	thematic analysis	Five themes were developed: pain is persistent and creates suffering, pain is subjective and poorly understood, pain is a biomedical problem that needs to be solved, pain is complex and more than a biomedical problem, and coping with pain is multi-faceted
Pujal i Llombart et al. [47]	Saude e Sociedade	Spain	This study aimed to present a critical, theoretical, and epistemological perspective on the analysis of chronified pain/fibromyalgia, and to develop a methodological tool that was in accordance with such perspective	1 woman with fibromyalgia	2 narrative interviews	case study, reconstruction of life story	The diagnoses can be described as meta-stories of life stories that weave in different variables until they describe a subjective phenomenological continuum that proceeds from the body to the social world and returns. The generalities of the psychosocial diagnoses of gender that were elaborated demonstrate a way of living chronified pain as disabling
Pujal & Mora [46]	Studies in Psychology	Spain	The authors proposed placing the concept of subjectivity at the heart of the discipline of psychology and understanding it from the theoretical perspective that overlaps between post-structuralism, psycho-dynamic theory and gender. The aim of the article was to support this proposal through a case study of chronic pain without an organic cause	1 woman with a fibromyalgia diagnosis	2 narrative interviews	case study; reconstruction of life story	New methodological instrument was developed: the psychosocial gender diagnostic, oriented toward capturing the dynamics of chronic pain. This methodology facilitated the integration of the connections between subjectivity, gender and health, while at the same time it problematized the excessive medicalization of modern-day life
Rice et al. [50]	The Journal of Pain	Canada	This study aimed to understand chronic pain among women who experience socioeconomic marginalization	27 women who are socioeconomically marginalized and have chronic pain	semi-structured face-to-face interviews	institutional ethnography	From women’s narratives, we identified 4 themes that speak to gender, chronic pain, and marginalization. These are gendered minimization of women’s health concerns, managing intergenerational poverty, living with violence and trauma, and gendered organization of family care
Schneider& Braungardt [52]	Der Schmerz	Germany	This study aimed to answer the question of whether there is a medicalization and pathologization of applicants (with chronic pain) for a disability pension	96 socio-medical assessments of applicants for a reduced earning capacity pension	documents	qualitative & quantitative document analysis	For around 24% of those assessed, the onset of the main physical complaints coincided with the onset of unemployment, while for 34% these symptoms only appeared after the onset of unemployment. The diagnoses from the psychiatric-psychosomatic area were also usually only made after the onset of unemployment
Sucaldito et al. [54]	North Carolina Medical Journal	USA	This study aimed to detail the health needs, priorities, and health care utilization of the Karenni, a Southeast Asian refugee community, in Forsyth County, North Carolina	101 Karenni adults	mixed-mode survey (online and in-person, qualitative and quantitative questions)	constant comparison and summarized into themes	Utilization of health care and public health services was low and impacted by individual- and contextual-level barriers, such as limited English proficiency and social determinants of health (e.g., lower levels of education and employment compared to state and national averages)
Wakelin [59]	Qualitative Health Research	UK	This paper aimed to combine lived experience of a pain condition without any apparent biological cause, to the wider issue of how we conceive and attend to embodied experience, shame, and power in qualitative health research	1 woman with chronic pain	written diaries, a notebook of pictures and free drawing, visceral memories of much of emotional experience	autoethnography	The implications from the study include personal emancipation, challenging the mind/body split, and emphasizing the interconnections between emotion and embodied experience, and the need for a pluralistic approach to treatment
Wallace et al. [60]	International Journal for Equity in Health	Canada	This study aimed to examine in depth the experiences of pain and discrimination and stigma across diverse marginalized communities in order to recommend equity-oriented healthcare approaches	36 participants, identify with one of three groups known to experience high levels of inequities and structural violence (indigenous group, LGBTQ2S group, two newcomer and refugee groups)	4 focus groups	thematic analysis guided by grounded theory coding techniques	Pain was entangled with and shaped by: social locations and identities, experiences of violence, trauma and related mental health issues, experiences of discrimination, stigma and dismissal, experiences of inadequate and ineffective health care, and the impacts of these intersecting experiences

## Results

We included 27 studies published between 1997 and 2024. Studies were conducted in the following countries, whereby one study was conducted in two countries: Canada (5), USA (4), UK (4), Australia (3), Norway (2), Sweden (2), Spain (2), Germany (1), Chile (1), Ethiopia (1), Switzerland (1), Jordan (1), worldwide (online) (1). The following data collection methods were used: semi-structured interviews (13), focus groups (6), online surveys (4), narrative interviews (2), participant observation (1), captured photographs (1), documents (1), Q-Sorting procedure (1), autoethnography (1). Table 2 provides a detailed overview of the study characteristics.

### Eight categories of social dimensions as an explanation for chronic pain

From our interpretation of the first and second order concepts and themes presented in the included studies, we developed eight categories of social dimensions as an explanation for chronic pain (third order). These are (1) *gender relations and gender inequity*, (2) *stigmatization, discrimination, and marginalization based on social identity,* (3) *adversities*, (4) *harsh living and working conditions*, (5) *high expectations regarding work or school*, (6) *cultural and religious beliefs and values*, (7) *loneliness*, and (8) *lifestyle factors*. In our analysis we focused exclusively on the study results related to our research question: Which social dimensions influence the development of chronic pain? This means that we did not analyze additional study results from the included studies. However, in Table 2 we provide an overview of the overall results of the studies from the perspective of the respective author(s). Table 3 provides an overview of the distribution of our eight categories of social dimensions as an explanation for the development of chronic pain across studies.

**Table 3:** Overview of the distribution of the eight categories of social dimensions across studies

Included studies	Gender relations and gender inequity	Stigmatization, discrimi-nation, marginali-zation based on social identity	Adversities	Harsh living and working conditions	High expectations regarding work or school	Cultural/religious beliefs and values	Loneliness	Lifestyle factors
Abudoush et al. [2]					x			x
Allen et al. [4]	x	x	x	x				
Arman et al. [5]	x		x	x	x		x	
Baum et al. [6]	x			x		x		
Black et al. [8]			x					
Brady et al. [9]		x	x			x		x
Brady et al. [10]		x	x			x		x
Campeau [13]		x	x				x	
Clottey et al. [15]	x		x	x				
Cousin et al. [17]		x				x		
Dassieu et al. [18]		x	x	x		x		
Eccleston et al. [19]								x
Furness et al. [23]	x		x	x	x			
Hallberg & Carlsson [25]			x	x	x		x	
Hervik et al. [27]			x					
Høie et al. [28]			x	x	x		x	x
Horment-Lara et al. [29]				x				x
Locher et al. [34]			x					
Mustafa et al. [40]	x			x				
Nigol et al. [42]		x	x	x				
Pujal i Llombart et al. [47]	x		x	x			x	
Pujal & Mora [46]	x		x					x
Rice et al. [50]	x		x	x				
Schneider & Braungardt [52]				x				
Sucaldito et al. [54]			x	x				
Wakelin [59]	x		x	x		x	x	
Wallace et al. [60]		x	x					

We present the eight categories we developed in detail with quotes from the studies we analyzed. To capture the breadth of the social dimensions, we inevitably have to forego some depth and cannot go into all 27 studies we analyzed in detail.

1. Gender relations and gender inequity

In developing our categories, we distinguished between gender as a category that primarily refers to biological sex and theoretical approaches that address *gender relations and gender inequality* as complex social constructs. Arman, Gebhardt, Hök Nordberg, and Andermo [5] and Cousin, Johnson-Mallard, and Booker [17] showed that women internalized gender-specific behavior, which was simultaneously expected of them by society. In particular, this expected behavior included caring for family and others, which led women to neglect their own needs. The neglection of own needs contributed to the development of chronic pain.

*The women described serving others too much and looking after other people’s welfare more than their own as being harmful to themselves. They were stuck in a life full of requirements, unable to find an escape. The women explained this as an internalized habit but also said society demanded it of them. (* [5]*, p. 776)*

Similarly, Mustafa, Ramana, MacNeill, Watt-Watson, and Einstein [40] who explored immigrant Indian women’s chronic pain experiences in Canada showed that gender relations, which are associated with an unequal distribution of housework and care work, contributed to women's overburdening.

*In general, when discussing painful body parts, participants talked about how they had little help with household chores, which they described as putting more of a toll on their own bodies than on those of other family members, particularly their husbands. (* [40]*, p. 7)*

According to Furness et al. [23] and Wakelin [59], experiences of pregnancy, childbirth and motherhood comprise a gender-specific characteristic that may contribute to women's overburdening and the development of chronic pain.

*Adjectives such as “difficult” (pregnancy, birth) and “traumatic” (labor) were used to reflect the psychological and physical impact of these events. In addition, there are broader impacts on a woman’s role and identity. Pregnancy and birth represent transition to a new life stage and the onset of demands of motherhood. (* [23]*, p. 3)*

Wakelin [59] reflected on the onset of her chronic pain in an autoethnography and saw a connection with her experience of birth and motherhood, which was accompanied by questions of identity and other situational events.

*[…] certainly contributed to my developing a pain condition. In addition, the intense situational factors at work at the time when the pain developed to do with motherhood, loss of identity, and a lost sense of competence and social belonging, all contributed to my not being able to recover. (* [59]*, p. 9)*

Pujal i Llombart, Mora, and Schöngut-Grollmus [47] argued that unequal gender relations within the framework of the expectation of gender equality led to women embodying this inequality in the form of chronic pain.

*Bodies lost in perpetual activity: the life trajectory of the perpetually active body, not just regarding physiological activity in general, but a body that is unlimited in the performativity of a feminine role, that becomes ambivalent due to the incommensurability of this role’s normative mandates within multiple contexts that are in play in times of equality (work, home, training, consumption). (* [47]*, p. 460)*

Similarly, Pujal and Mora [46] emphasized that the claim to gender equality is unredeemable in the context of inequality for women and results in an incorporation in the form of pain becoming chronic.

*The corporal dimension of DC/FM [Fibromyalgia] finds a corresponding psychic that has to do with the impossibility of a self that manages the ambivalence of roles in the search for a subject within this multiple context that contemporary women live. (* [46]*, p. 222)*

Baum et al. [6] pointed to another dimension of gender relations, a strict gender segregation in some societies. Such gender segregation was observed by Baum et al. [6] in the Somali region of Ethiopia and resulted in women facing difficulties in accessing healthcare and reporting pain because health workers were predominantly male. These difficulties in accessing healthcare and reporting pain could lead to the development of chronic pain.

*Some women, however, did describe uneasiness with regard to male health providers and difficulty reporting their pain to others. Several men mentioned that women felt ashamed or feared further health interventions or diagnostics if they informed others about their pain. From their point of view this may result in additional check-ups performed by male health providers. (* [6]*, p. 12)*

(2) Stigmatization, discrimination, and marginalization based on social identity

Neglecting appropriate treatment of illnesses can foster chronicity. In addition to gender, certain other social identities and locations can lead to stigmatization and discrimination within the healthcare system and limit access to appropriate healthcare. Participants in the study by Allen, Murphy, Kiselbach, VandenBerg, and Wiebe [4] reported that they were not listened to and that this experience of discrimination led them to refrain from seeking medical care again. Participants in this study were female survival sex workers in Vancouver's downtown eastside.

*’I don’t wanna go back through not being listened to again, do you know what I mean there, right?... If you can reject it first before somebody rejects you, it’s easier to shut it down. It’s that wall you put up, right?’ (Participant 4) (*[4]*, p. 4)*

Participants in the Brady, Veljanova, & Chipchase, 2019 study in Australia reported being stigmatized because of their belonging to a visible minority. Participants were people who identified themselves as Mandaean, Assyrian, or Vietnamese. They felt that doctors did not believe that they were in pain.

*Specifically, participants reported the stigma of belonging to a visible minority during encounters with health providers for chronic pain and the fear that “the doctor doesn’t believe one thing I say to her....She probably feels that I want to go on the pension and that’s it” (A2, p40). (* [10]*, p. 441)*

Similarly, participants in the [42] study reported a link between their experience of stigma and their pain. Participants in this study were people belonging to the Nepali-speaking Bhutanese community in Australia.

*Participants noted that stigma and judgement from others could also result in pain. One participant said that they believed the doctor’s role was not simply to prescribe medication, but also to hear the patient’s experiences and concerns. (* [42]*, p. 0)*

At the same time, studies showed that stigmatization and discrimination based on social identity and location in themselves contribute to poor mental and physical health, including chronic pain. This means that the chronic pain in this case is not attributed to poor access to healthcare, but to the experience of negative discrimination and stigmatization itself. This is referred to as a kind of “secondary pain” [4]. Participants felt discriminated against and stigmatized based on various social identities and locations: having a certain ethnic background (e.g. first nation) [4, 60], having a substance dependence/using drugs [4, 18], being a sex worker [4, 18], race [17], being a refugee [42, 60], being new or a stranger in another country [13, 42, 60], identifying as LGBTQ2S [60], belonging to a visible minority [9, 10].

Participants in the [13] study related the starting of their pain to migration and being new in another country. Participants were women who had fled from Somalia to the United States. These experiences raised a feeling of isolation, loneliness, disorientation, and marginalization.

*Many participants traced their experience of chronic pain to the experiences of migration and being strangers in a new land, surrounded by people speaking in an unfamiliar language, filling out paperwork for unknown institutions and settling families into temporary living quarters. Oftentimes, participants’ symptoms map onto experiences of rupture and relocation. (* [13]*, p. 100)*

Similarly, in the [60] study in Canada, participants associated their chronic pain condition with their social identity status as newly arrived refugees and experienced social isolation, among other things, as a result.

*The social identity status of being a refugee newcomer with its attendant consequences of social isolation was also identified as a cause for the advent of chronic pain. (* [60]*, p. 5)*

(3) Adversities

Numerous studies showed a connection between *adversities* participants had experienced and the development of chronic pain. In some cases, participants themselves attributed the adversities to their chronic pain [8, 10, 23, 27, 42, 47, 50, 60]. Brady et al. [10] reported that.

*[…] the Mandaeans and Assyrians portrayed an intersection between life in their homeland, forced migration, and their current pain status. Both communities recounted experiences of life in a war-ravaged country and the associated physical and psychological consequences for their health. (* [10]*, p. 437)*

The participants in the [42] study also experienced forced displacement, which was accompanied by the loss of their homeland as well as physical and psychological violence. Participants attributed their chronic pain to these traumatic experiences.

*Participants discussed how traumatic memories could trigger feelings of pain, lead to a sense of disconnection from the body and impact the soul. (* [42]*, p. 0)*

Participants in the [27] study in Norway were people who suffered from chronic headaches. In some cases, they also attributed their chronic pain to traumatic experiences.

*Ten participants believed that trauma was the reason for their symptoms. Four participants reported headaches after physical trauma and six referred to psychological events. (* [27]*, p. 707)*

The study by Wallace et al. [60] in Canada included three groups of people known to experience high levels of inequities and structural violence. These were an indigenous group, an LGBTQ2S group and two newcomer and refugee groups.

*[…] in all groups many associated their chronic pain with personal experiences of trauma and related mental health issues. (* [60]*, p. 5)*

Rice et al. [50] included people in Canada who were socioeconomically marginalized and suffered from chronic pain and found the same result: Participants associated the onset of their pain with traumatic life events.

*Lisa […]: When you’re in threat and your mind figures you can’t get out, your body is going to do whatever it can to help you survive. And it got me through those 26 years of significant abuse, but the problem is it took a very big toll on my body physically. They say that it was related to the CRPS [complex regional pain syndrome], but in hindsight, I think it was more related to the trauma that was being stored in my body. (* [50]*, p. 7)*

Several studies examining the causes of chronic pain in people diagnosed with fibromyalgia also reported that participants associated the onset of their pain with negative life events such as intimate partner violence, sexual abuse, and child neglect [8, 23, 47].

*Another common response was the belief that emotional trauma/stress caused their fibromyalgia. While some participants provided more general answers of “emotional stress” or “trauma”, others specifically identified the stress of being victims of intimate partner violence as the trigger for their symptoms. (* [8]*, p. 3)*

*“I was sexually abused as a child, my dad and husband were both alcoholic and violent, so many years of stress, pain, anger, resentment, fear, I think, have contributed to fibromyalgia.” (Peggy, F, 46, S94, D06) (* [23]*, p. 3)*

Pujal i Llombart et al. [47] reconstructed a life story of a woman with fibromyalgia who also associated an adverse event in her life with the onset of her chronic pain.

*[…] my grandfather died – he was the only person who sometimes came to find me and took me to the theater to see ‘Els Pastorets’ and things like that. And, for me, my grandfather’s death – he died at home and I watched him die… affected me so strongly, that I began to have dreams at night and crying and ever since then I started to have pain, and I had pain. (* [47]*, p. 457)*

In other cases, the study authors attributed the chronic pain condition to experienced adversities. Hallberg and Carlsson [25] included women with fibromyalgia. They related the following experienced adversities to the development of chronic pain of their participants:

*[…] alcohol abuse, violence, and psychiatric diseases in one or both parents, were described by several women. Two women had been assaulted for many years by their stepfather and partner, respectively. (* [25]*, p. 98)*

Campeau [13] recognized that.

*[w]hen participants spoke of their pain, many also spoke of their lost homes in Somalia. (* [13]*, p. 99)*

In the [28] study, it was not the authors and not the people suffering from chronic pain who linked their condition to adversities in life, but a third party. These were school nurses (SN) who worked with adolescents suffering from chronic pain.

*One SN talked about a girl who had a stomach ache for three years, even though she was examined at the hospital several times without result. No one linked her pain to having lost a close family member. However, when she finally joined a grief group, the stomach ache disappeared. (* [28]*, p. 4634)*

Similarly, in the [34] study, a third party linked an adolescent's chronic pain to adversities in the adolescent's life such as family or school problems. The third-party members in this study were pediatricians, who were asked to explain the causes of an adolescent's chronic pain using a case vignette. However, the vignette did not mention any adversities the adolescent had experienced.

*A number of quotations suggested that pain was caused by social circumstances, such as family or school problems. Examples: “Identify stressors and tension in the family, at school and with peers.” (* [34]*, p. 4)*

(4) Harsh living and working conditions

A further social dimension related to contributing to the development of chronic pain were *harsh living and working conditions*. The harsh working conditions were characterized by poor or irregular pay, insecurity due to informal employment without workers'rights, and stressful and monotonous tasks, which overall contributed to chronic pain conditions as Hallberg and Carlsson [25] showed with their study with women with fibromyalgia.

*The work situation was often described in negative terms, e.g. as a monotonous and strenuous job, sedentary work, stressful work, and no opportunities to influence their work. (* [25]*, p. 99)*

The Horment-Lara, Lüttges-Sciaccaluga, Espinoza-Ordóñez, and Aliaga-Castillo [29] study in Chile included women with non-specific chronic low back pain. The participants cited too much work and starting work too early as reasons for the development of chronic pain.

*“Too much work. I worked my whole life. From twelve years old I worked a lot. I think that carrying so much weight, that affected me little by little” (Dalia). (* [29]*, p. 3)*

Excessive work hours were highlighted as a cause of chronic pain in the studies by Sucaldito, Meh, Rhodes, and Daniel (2024) and Mustafa et al. [40]. Sucaldito et al. [54] focused on people from the Southeast Asian Karenni refugee community living in the US and concluded that.

*[…] concerns about physical pain were attributed most often to mental health concerns (e.g., “anxiety and stress lead to physical aches”) or work (e.g., “chronic body pain from long time work and even after stopping working”). (* [54]*, p. 291)*

Mustafa et al. [40] included in their study Indian women who had migrated to Canada. They showed that the working conditions of the women in Canada deteriorated significantly. Longer work hours, longer commutes and other types of work contributed to the development of chronic pain among the participants.

*In Canada, they worked long hours—in some cases, 12-h shifts—washing dishes and standing on their feet, whereas in India they either did not have outside employment or, if they did, it was an office job with fewer hours. Employment outside the home as well as the types of jobs requiring long hours of standing resulting from immigration became sources of pain. (* [40]*, p. 8)*

Baum et al. [6] also emphasized that harsh daily living and working conditions influenced the development of chronic pain of pastoralists living in rural areas of Ethiopia. However, authors and participants themselves did not report that this was the sole cause of their chronic pain.

*Most participants and particularly pastoralists from rural areas described pain as something normal everyone must face. Pain was part of their daily life and, therefore, something they had learned to live with. The majority of interviewees described loading animals, harvesting or fetching and carrying water over long distances as strenuous activities that were part of their daily routines. (* [6]*, p. 8)*

Dassieu et al. [18] identified the harshness of the drug use environment and precarious working conditions as sources of chronic pain in people who use illegal drugs. These were the participants in their study and in many cases, they lived on the street.

*Others explained the onset of pain by the wear and tear on their body caused by life on the street: It's not surprising that I have all this pain. I spent … I don't know any more if it's eight years or 10 years, on the street. Either 8 years, or 10 years, I don't remember. And then, I was sick. I spent some winters out in the cold; it wasn't easy. (* [18]*, p. 3)*

In the context of economic difficulties, Clottey, Scott, and Alfonso [15] showed that housing was insecure and often only available to participants in unpleasant areas and dirty houses. In these neighborhoods, health infrastructure was often poor and long distances to medical facilities were unaffordable for people with economic difficulties and limited time resources due to many daily obligations such as childcare, paid work, and housework.

*The caregivers faced a double bind related to their own health needs. Their health problems were exacerbated by the strain of caregiving, but caregiving also made it more difficult to access care, both because of finances and logistics. (* [15]*, p. 6)*

Allen et al. [4] also pointed out the connection between economic difficulties and the chronicity of pain, because.

*[…] prescribed lifestyle changes and therapies for chronic pain were described as inaccessible or unaffordable. (* [4]*, p. 4)*

Rice et al. [50] even showed that poverty and its effects on pain development can be passed on between generations.

*For example, Zinnia (70s, British Columbia) describes the effects of intergenerational poverty in her life and explains that this has carried forward in that her adult son struggles with health and finances as well. She states that she feels guilt for passing on her chronic pain to her son, who is unable to work due to his pain. (* [50]*, p. 6)*

Several studies [5, 23, 29, 40, 47, 60] emphasized that women in particular faced a double burden when they take sole or primary responsibility for family life and simultaneously are employed elsewhere. This included caring for children, sick relatives (e.g. partners, elderly relatives), cleaning the house, preparing meals, and resolving family conflicts.

*“Stresses associated with caring were commonly mentioned: “My husband had a stroke abroad while on holiday with our three children, aged 6, 7 and 10. My husband was 30 years old. He has been disabled since then.” (Sharon, F, 52, S96, D13) (* [23]*, p. 4)*

These studies emphasized that the life situation of the participants has been under chronic stressors due to too many tasks. In these situations, participants felt they were no longer able to manage life without rest and support.

*Giving care and having responsibility for the welfare of others became a constraint. This was experienced as causing negative consequences for the women’s health. (* [5]*, p. 776)*

When rest and support in life were not available, these circumstances contributed to the development of chronic pain. The dual responsibility for household and care duties as well as for employment can also be taken by grandparents for their grandchildren as Clottey et al. [15] reported in a study they had conducted in the United States.

*Unstable and insufficient money flow added to their vulnerability to poor health outcomes, both directly and indirectly. In some cases, grandmothers reported foregoing doctor visits or the purchase of their medications because of expenses related to their grandchildren. In addition, lack of childcare support made health appointments and hospitalizations logistically difficult. (* [15]*, p. 8)*

An unstable financial situation combined with a lack of time for self-care due to too many obligations can lead to poor health and promote chronic conditions such as chronic pain. Hallberg and Carlsson [25], Høie et al. [28] and Pujal i Llombart et al. [47] showed that also children sometimes had more responsibility than usual for their age due to exhausting family situations. The greatest burdens included the family's difficult financial situation and the mother’s or father's alcohol or drug abuse, which meant that the children had to take on parental responsibilities.

*It was obvious that most women had had a high degree of responsibility early in life, e.g. responsibility for younger brothers and sisters and/or housekeeping in their primary family. Several women had started work early in life, e.g. after a full day at school in their early teens, in order to earn money. (* [25]*, p. 98)*

Based on a document analysis of socio-medical assessments of applicants for a reduced earning capacity pension in Germany, Schneider and Braungardt [52] showed that the social and psychological consequences of long-term unemployment contributed to the chronicity of pain.

(5) High expectations regarding work or school

We subsumed high demands on oneself under the category *high expectations of work or school*. The effects of high expectations of work or school lead in many cases to stress and too few recovery periods for the participants, which contributed to the development of chronic pain. Arman et al. [5] emphasized that.

*The women experienced a sense of having to be strong and endure for long periods without seeking sick leave. (* [5]*, p. 776)*

Hallberg and Carlsson [25] also pointed out that the women who took part in their study placed particularly high demands on themselves.

*All women shared high personal standards and had always felt strong internal rather than external demands to be capable, effective, caring, and friendly. They all seemed to drive themselves harder than people in general. (* [25]*, p. 99)*

Similarly, Furness et al. [23] showed that participants put pressure on themselves to cope with high workloads and not take breaks, which contributed to their chronic pain condition in the long run.

*”Work stress was not always the result of external demands: our participants acknowledged putting pressure on themselves, admitted “over-working,” or being “very stubborn and [carrying] on through relapses when I maybe should have rested” (Theresa, F, 63, S70, D93). (* [23]*, p. 4)*

The participants in the [2] study recognized for themselves a connection between increased pain and the demand to meet, for example, deadlines at work.

*Participants realized their pain increased with specific postures at work, mainly when they had a deadline (P49). One female student participant expressed how her routine at the study workstation was connected to the pain. “I feel that the pain increases when I work a lot on a computer or when I am stressed, I mean, lots of things to do on that day.” (* [2]*, p. 5)*

High demands on oneself are also influenced by social and external expectations, as the school nurses in the [28] study emphasized when talking about adolescents with chronic pain. In this study, high demands on oneself related both to performance at school and in the social networks with peers.

*All SNs [school nurses] in this study associated adolescent pain with high expectations, both from the adolescents themselves and from their surroundings: “To always be the best is demanding. A lot of young people experience the pressure of always being at their best.” SNs gave multiple examples of how the parents of the adolescents expected high grades and put pressure on their children. (* [28]*, p. 3)*

(6) Cultural and religious beliefs and values

A common pattern across studies that we mapped to the dimension of *cultural and religious beliefs and values* ​​was not telling others about pain and feeling unwell while simultaneously presenting oneself as strong to others. The specific reasons for this stoicism were somewhat different, but the consequences were the same: delay in pain treatment and chronicity of pain. In her autoethnographic reflection, Wakelin [59] came to the conclusion that her stoicism contributed to the chronicity of her pain. At the same time, she concluded that stoicism towards the illness is socially constructed, and gender related.

*My own history of stoicism and avoidance of pain interacted with ideas both about women and more generally about society’s shame around illness, to add to a sense of myself as fragile and unworthy. (* [59]*, p. 3)*

Baum et al. [6] showed that stoicism was particularly present in relation to gender, age and marital status. They explained this by the participants'fear of not finding a spouse if others knew about their pain since pain is associated with stigma.

*Particularly in rural areas, stoicism or impassiveness was a topic–associated with age and marital status. Younger, unmarried adults and especially women were more inclined to bear or hide their pain, since they were more worried about stigma. (* [6]*, p. 11)*

In the study by Cousin et al. [17], fear of discrimination due to expressing pain and accepting or asking for help was also present. The authors demonstrated a connection between gendered and racial expectations, which contributed to the image of the “strong black woman” ([17], p. 3) and contradicts the expression of pain and the request for help.

*Older Black women often presented the appearance of being strong and a family pillar as a method of self-preservation as mother, sister, caregiver, friend, and woman: “You got to be the strong person. Yep. No, it’s not easy” (W1). Women did not want to appear sick, “crippled,” or in pain and found ways to hide physical joint deformity and prevent the appearance of being disabled. (* [17]*, p. 7)*

The desire to avoid the appearance of disability due to pain mentioned by Cousin et al. [17] was also present among the participants in the Dassieu et al. [18] study. The participants were people who used illicit drugs (PWUDs) and they presented themselves as “being tough” ([18], p. 5), which was relevant to them within their social environment.

*PWUD's attempts to maintain the appearance of “being tough” foster the clinical invisibility of their pain. The desire to tolerate pain while maintaining everyday activities led some participants to acknowledge the pain as a problem only several weeks or months after its onset and escalation. (* [18]*, p. 5)*

The presented studies showed that the invisibility of pain due to cultural and religious values and beliefs ​​such as stoicism and the necessity to appear strong and healthy contributed to pain becoming chronic. Brady et al. [9, 10] also contributed the influence of a collectivist perspective that gives priority to the collective over the individual. By collective they referred to an ethnic community as well as to the family.

*Quotes presented in *Table 2* reflect the traditional, collectivist views of all three communities. As such, the identity of the individual is entwined within their role in the family and wider community. Accordingly, the needs of an individual experiencing pain are viewed second to the needs of the family and wider community. (* [9]*, p. 5)*

This value of ​​the collective can lead to people being unable to adhere to recommended pain management and neglecting their individual treatment in favor of the interests of the community, which may contribute to the pain becoming chronic.

*Further, management approaches, such as activity pacing and exercise, were evaluated by participants against their role priorities and, when incompatible, were discarded. (* [10]*, p. 437)*

(7) Loneliness

The category *loneliness* refers to a practical and emotional dimension. Both dimensions can contribute to the chronicity of pain. Following Arman et al. [5] and Wakelin [59], this includes the sole responsibility for organizing family life and the lack of opportunities to share personal problems and concerns about oneself or the children with others.

*An implicit loneliness in everyday life, including responsibility for the family, appeared to be a subtext in women’s lived experience. Problems were endured without a supporting network. “We didn’t have any support from relatives anywhere—no-one’s close by. So, it’s always been just me...” Besides the practical challenges of everyday life, the women also experienced emotional loneliness. (* [5]*, p. 775)*

Similarly, Wakelin [59] links her emotional loneliness and sole responsibility for the children without a supportive social network to the origins of her pain.

*Each time I recovered, until on one occasion the antibiotics did not work, and the pain stayed permanently, although the infection itself cleared. That time I felt a particularly strong sense of loneliness, of not being understood. I felt despair and fear. I had not gone back to work, my time was dedicated to my children, and unlike with my first child, I did not have a circle of friends with whom to share the experience. (* [59]*, p. 2)*

Campeau [13] showed that the participants experienced loneliness because they had fled their home country and did not know anyone in their new place. The participants themselves also linked their chronic pain to their experiences of loneliness.

*”I never had pain like this in Somalia. There, I was healthy, I worked hard. My headaches started here. When I was here, there were less Somalis, and I knew no one. And then they (headaches) never went away.” (Waris, aged 56 years, 16 years in the USA)” (* [13]*, p. 100)*

Hallberg & Carlsson, [25] study showed that the participants experienced loneliness due to rejection by their parents in childhood. For some of the participants, this emotional pain extended into adulthood and led to chronic physical pain.

*One woman said, ‘I have never felt that I was loved by my mother. She could not understand me.[…]’ (* [25]*, p. 98)*

The emotional pain experienced as an adult also contributed to the participants'chronic pain, as Pujal i Llombart et al. [47] showed by reconstructing the life history of a woman with fibromyalgia.

*Her own family, in which she had two children – a girl and a boy (that he legally rejected during the pregnancy, another one of the many acts of family abandonment), have made her into a lonely person for more than 40 years, mainly emotionally, both in her maternal role and as provider, as she has always had two or three jobs, given the time of women’s liberation and “equality” that she had to live (the social model of transition of gender and multi-tasking). (* [47]*, p. 459)*

Høie et al. [28] showed that adolescents experienced loneliness because they were unable to cope with the high demands of parents, school or peers. This led to feelings of inadequacy and contributed to the chronicity of pain.

*SNs [school nurses] also said that adolescents compared themselves with others and could feel unsuccessful or left out, giving them mental pain in terms of loneliness and depression. One of the SN told: “Sometimes, with those I know better…I understand that they have extremely high demands on themselves...or from their parents…first, they talk about their daily physical pain. Sometimes, someone also touch upon issues such as being sad, anxious and lonely.” (* [28]*, p. 4)*

(8) Lifestyle factors

We identified *lifestyle factors* as an eighth social dimension influencing the chronicity of pain. Several studies have highlighted various behaviors that can be defined as lifestyle factors. Both the authors and the participants linked lifestyle to the development of chronic pain. For example,

*[…] lifestyle factors such as diet, obesity, “not exposing enough to sunlight” (P3), “cold weather” (P13), and “lifestyle stress” (P12) had an effect on CP [chronic pain]. Food quality or the type of food influenced the development of CP in a few participants. (* [2]*, p. 5)*

The type of diet was also identified by one participant in the [10] study as a factor influencing the development of her pain.

*She attributed her physical vulnerability to the interaction between adherence to ethnoreligious dietary customs, cultural expectations of a woman’s behavior […] (* [10]*, p. 441)*

One participant in the [29] study explained the development of her pain with a previous behavior that can be subsumed under the category lifestyle factor.

*“I used to wear high heels all the time. Women have no idea what terrible damage that does to your back. Those heels just tear your back up over time. At the time you don’t notice, but after years and years you feel it in your back” (Ester). (* [29]*, p. 3)*

In the study by Høie et al. [28], school nurses from a third-party perspective linked a certain lifestyle of adolescents to the development of different forms of chronicity of their pain.

*The SNs also talked about adolescent inactivity and extensive use of social media and connected this to the fact that many were bothered with headache, neck, back, and shoulder pain. Moreover, they suggested that young people could not put their cell phones or computers away, causing them to go to bed very late. The result was that a lack of sleep made it hard to get up in the morning, they were tired, and headaches increased. (* [28]*, p. 5)*

Less sleep due to lifestyle was also mentioned by Pujal and Mora [46]. They reconstructed the life story of a woman with fibromyalgia who worked in the film and television industry.

*This glamorous work confirms her self through the public world, to the point that she completely ignores her body, she hardly eats or sleeps, and the rest of her life: her partner and the maternal project they had together. (* [46]*, p. 220)*

While Pujal and Mora [46] strongly emphasized that people ignore signs that their body needs rest due to their stressful lifestyle, Eccleston, Williams, and Rogers [19] pointed out the aspect that people with pain partly make pain part of their lifestyle. Participants in their study strongly supported the statements “Often chronic pain is less a'condition'and more a way of behaving"and “For some people, chronic pain becomes a way of life” ([19], p. 704). This means that when patients are in pain, they may learn certain behavioral patterns and types of lifestyles that may worsen their problem and cause the pain to become chronic.

## Discussion

Despite the frequent recourse to the biopsychosocial model of pain in chronic pain research, the social dimension has hitherto been neglected as an explanatory approach for research on chronic pain onset and progression. Our meta-ethnography confirmed that this gap exists. After reviewing the 560 abstracts and 79 full texts of our database search, we finally found only 27 qualitative studies that addressed social dimensions as an explanation for the development of chronic pain in addition to psychological and biomedical explanations. Despite this small number of studies included, our analysis showed great heterogeneity in the social dimensions that contribute to explaining the development of chronic pain. We developed eight categories of social dimensions, which are third-order constructs that partly correspond to and partly go beyond the second- and first-order constructs of the included studies.

Overall, it is noticeable that gender represented a particularly prominent category in our sample as an explanation for the development of chronic pain. This result may not be surprising. After all, quantitative epidemiological studies have already shown that women suffer from chronic pain more often than men [7, 14, 31], and also girls more often than boys [24]. Although quantitative studies show that women are more frequently affected by chronic pain, they cannot explain these relationships in depth. In addition, gender is often understood in a categorical sense that mainly refers to biological sex. Scholars criticized this “conflation of gender with women” ([26], p. 1713), which can be observed particularly in research and policy practice in the health sector [3, 16, 26]. Scholars have highlighted the limitations of research designs that emphasize predetermined classifications (e.g., male and female) or favor a single category (e.g., sex) or even a fixed constellation of variables (e.g., gender) in a contextual analysis [26]. Due to the pre-determination of certain social categories, a focus is made that otherwise would not necessarily manifest. The complex interrelationships between different social dimensions would thus be given too little attention.

In our sample the research interests of 12 of 27 studies addressed female participants and as a result only included participants who identified themselves as female. This selection of research interests and participants partly explains why gender represents a particularly prominent social dimension in the development of chronic pain in our analysis. At the same time, this focus by the authors of studies in our sample shows that they were building on the existing knowledge that women and girls are more frequently affected by chronic pain to obtain more in-depth understanding of this prevalence. Our analysis of these studies showed more profound explanations between gender as a social construct and the development of chronic pain. Accordingly, we have conceptualized the category *gender relations and gender inequality* to point out that gender is a complex social construct that is shaped by societal norms, socialization processes, forms of expression, and behavior [16, 26]. Gender, understood in this dynamic and relational sense, intersects with some of our other categories of social dimensions, especially harsh living and working conditions, adversities, and cultural and religious values ​​and beliefs. Our analysis showed that in ten studies gender was explicitly understood as a complex social construct and was used to explain the development of chronic pain [4–6, 15, 23, 40, 46, 47, 50, 59]. For example, obstacles to women's access to early and appropriate treatment included gender segregation in society combined with a shortage of female doctors, and the double burden of care work and employment, which affects women more often than men. [50] identified the intersection of being a woman and the associated expectation to undertake care work with living in socioeconomically marginalized conditions as a cause of pain chronicity. Mustafa et al. [40] showed how the intersection of being a woman and the associated expectation to care for children and family with migration, which led to insecure and harsh working conditions, caused chronic pain. Campeau [13] illustrated how the intersection of being a woman and a refugee with the experience of an unfamiliar language and institutional arrangements can lead to feelings of isolation, loneliness and disorientation that can cause pain chronicity. These results highlight the intersection of social dimensions as explanatory approaches for the development of chronic pain that go beyond a categorial understanding of gender as merely biological sex.

Scholars have further highlighted that lack of early intervention and appropriate treatment contributes to the development of chronic pain conditions and that social inequalities and marginalization influence both the prevalence of chronic pain and its treatment and management [22, 35]. It is recognized that socioeconomic and neighborhood disadvantages impair access to pain treatment and the ability to manage pain [35, 56]. It is also recognized that racial and ethnic discrimination or stigma in the healthcare system interferes with appropriate and timely pain management [12, 22, 38, 45]. Several of the studies included in this meta-ethnography showed how the intersection of different social dimensions influenced delayed intervention and appropriate treatment. Brady et al. [9, 10] and Nigol et al. [42] explained barriers to accessing healthcare facilities in Australia by experiences of racial discrimination by medical staff who disbelieved those affected because they belonged to a visible minority group. Because of these experiences of discrimination, those affected tried to avoid healthcare facilities, even when in pain. The experience of discrimination in the healthcare system in the form of disbelief of having pain can, as Allen et al. [4] emphasized, also result from the intersection of being a woman and a sex worker. Other studies [15, 17] illustrated how the intersection of female and racialized experiences in US society constitutes the so-called stoic identity of ‘strong black women’. This identity attribution explained why Black women in the US tend not to express their pain, even when they are in pain, which is accompanied by a reluctance to seek healthcare help for their pain. The reluctance to seek medical help for their pain can, as Dassieu et al. [18] argued, also be understood as a consequence of the intersection of using illicit drugs and living in poverty, since harsh living conditions, such as being homeless, can foster a stance of expressing oneself as ‘tough’. Thus, the intersection of harsh living and working conditions, and experiences of marginalization, discrimination, and stigmatization due to different social identities can hinder access options to adequate and early pain treatment and management, which influence the genesis of chronicity of pain. In addition, the included studies demonstrated that experiences of physical, psychological, or emotional stress, loneliness and certain lifestyle factors can influence the development of chronic pain.

In summary, this meta-ethnography showed that exploring social dimensions as an explanatory approach to chronic pain onset and progression can be more deeply understood by analyzing qualitative studies, as individual experiences of chronic pain are always embedded in particular socio-structural contexts. We derived some implications for policy and clinical practice from the insights of our meta-ethnography.

## Implications for policy and clinical practice

- Female, gender-sensitive, and trauma-informed health professionals should be available
- People's narratives and experiences should always be taken seriously
- More interdisciplinary and holistic approaches should be encouraged, especially including more social workers in medical teams
- Medical education should integrate gender and discrimination sensitivity training
- Health care services need to be available in socio-economically disadvantaged neighborhoods
- Childcare options within health care centers can assist people with care responsibilities to take part in physiotherapy, rehabilitation sports and other chronic pain management programs
- Programs to combat poverty, such as increased social housing construction, are needed
- Anti-discrimination and -stigmatization programs should be initiated or strengthened

## Limitations of the study

Our review has several limitations. One limitation is that we only considered qualitative studies that were published in English or German. There may be other relevant studies in other languages. A second limitation is the selection of databases. Even though we have already included three large and thematically relevant databases in our search, an expansion of the databases could lead to further suitable studies for our inclusion criteria. Both extensions could have led to the inclusion of further suitable studies and thus potentially to the development of additional social dimensions that cause the onset and progression of pain chronicity. Furthermore, more than half (16) of the 27 included studies were conducted in Anglo-American countries (Canada, USA, UK, Australia), eight in European countries, and only three in non-European and non-Anglo-American countries, and one worldwide (online). This must be viewed as a limitation of the social dimensions examined as explanatory approaches for the onset and progression of chronic pain. However, seven of the studies conducted in the Anglo-American or European region focused on people with a migration or refugee history from non-European or non-Anglo-American countries, which in turn contributed to a diversity of the included perceptions and experiences of people with chronic pain.

## Conclusions

There are only a few qualitative studies that explicitly focus on the social dimension as an explanation for the onset and progression of chronic pain. However, these studies show a wide variety of influencing categories within the social dimension. This means that the prevention of chronic pain should address these issues in addition to the biomedical and psychological aspects. This in turn implies that the responsibility for the prevention of chronic pain onset and progression cannot lie solely with the health system, but that social policy in particular could play a greater role in the prevention of chronic pain by tackling marginalization. This means addressing poverty e.g. through housing programs, the provision of secure jobs and childcare facilities, and improving health infrastructure in remote areas and disadvantaged neighborhoods.

### Website

Pain IAftSo. IASP Taxonomy—IASP. 2015 [cited 2024 September 26]. Available from: http://www.iasp-pain.org/Education/Content.aspx?ItemNumber=1698&navItemNumber=576.

## Data Availability

No datasets were generated or analysed during the current study.
